# Potential mechanisms by which impaired ketogenesis links metabolism to T‐cell dysfunction in patients with severe COVID‐19

**DOI:** 10.1002/mco2.178

**Published:** 2022-09-30

**Authors:** Bin Wang, Lei Zhang, Fangfang Zhou, Long Zhang

**Affiliations:** ^1^ MOE Laboratory of Biosystems Homeostasis & Protection and Innovation Center for Cell Signaling Network, Life Sciences Institute Zhejiang University Hangzhou P. R. China; ^2^ Department of Orthopaedic Surgery The First Affiliated Hospital of Wenzhou Medical University Wenzhou P. R. China; ^3^ Institutes of Biology and Medical Science Soochow University Suzhou P. R. China

1

In a recent study published in *Nature*, Karagiannis et al.^1^ reported that severe acute respiratory syndrome 2 (SARS‐CoV‐2)‐induced T‐cell dysfunction is associated with an attenuated increase in β‐hydroxybutyrate (BHB) in the circulation. This suggests that impaired infection‐induced ketogenesis may occur in patients with SARS‐CoV‐2‐induced, but not influenza‐induced, acute respiratory distress syndrome (ARDS). Supplementation of BHB through a ketogenic diet (KD) or delivery of BHB as a ketone ester drink reduces mortality in mice infected with SARS‐CoV‐2, which provides a new insight into KD‐based coronavirus disease (COVID‐19) treatment (Figure 1).

**FIGURE 1:**
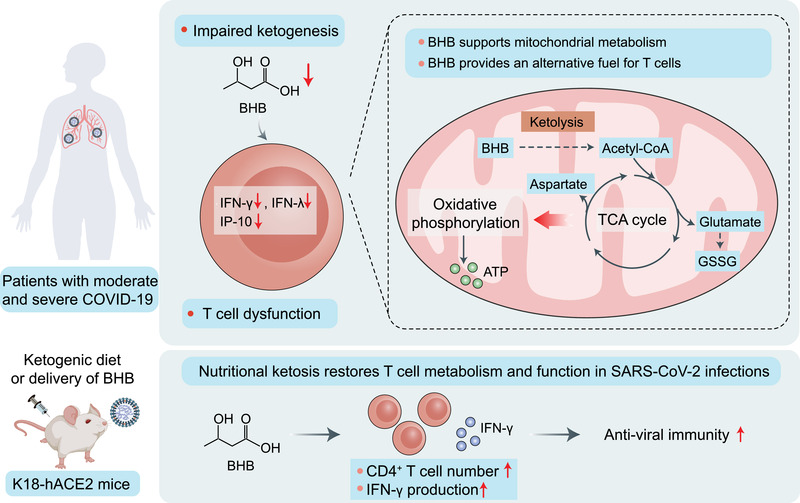
Potential mechanisms by which impaired ketogenesis links metabolism to T‐cell dysfunction in patients with severe COVID‐19. The current study^1^ demonstrated that β‐hydroxybutyrate (BHB) acts an alternative carbon source to fuel oxidative phosphorylation and the production of bioenergetic amino acids (aspartate and glutamate) and oxidized glutathione (GSSG) for T cells, thereby promoting T‐cell responses in pulmonary viral infections. Impaired ketogenesis and BHB production in patients with moderate and severe COVID‐19 may be at the root of the metabolic dysregulation and defective effector function of T cells. Elevating serum BHB concentration by feeding a ketogenic diet or supplementing with ketone ester in drinking water restores CD4^+^ T‐cell metabolism and function in viral infections and reduces the mortality mice infected with SARS‐CoV‐2. IFN, interferon; IP‐10, interferon‐γ‐induced protein‐10

During starvation, fasting, or certain pathological conditions (such as diabetes mellitus and acute infection), human metabolism is forced to switch the nutrient supply. Ketone bodies (including acetone, acetoacetic acid, and BHB) produced by the ketogenesis of hepatocytes from free fatty acids are an important alternative fuel for peripheral organs and support host survival in response to infection. Accumulating evidence suggests that ketone bodies exert a direct effect on immune cells. For example, Goldberg et al.^2^ reported that feeding mice a KD for 1 week provided protection from IAV infection by increasing the survival of the mice along with reducing the viral load in the lungs and bronchoalveolar lavage fluid relative to mice fed a normal chow diet. By characterizing the immune response in the lungs and using mice lacking γδ T cells, they identified that KD promoted the expansion of γδ T cells in the lung. In addition, the treatment of macrophages with BHB prevents NLRP3 inflammasome‐mediated inflammation.^3^ Recently, Zhang et al. reported that ketogenesis‐generated BHB is an epigenetic regulator of CD8^+^ T‐cell memory development.^4^ The COVID‐19 pandemic is caused by SARS‐CoV‐2 and has posed serious threats to public health and the global economy. The two main immune cell groups that the human immune system clears the SARS‐CoV‐2 invasion are CD4^+^ type 1 helper T (Th1) cells and CD8^+^ cytotoxic T cells, which are produced by cytokines (such as interferon [IFN]‐γ) or directly killed infected cells do their job. Cellular metabolism and mitochondrial function are major determinants of T‐cell activation and function. In the context of the current COVID‐19 pandemic, the question has been raised as to whether ketogenic metabolism affects the antiviral immune response to lung infection in patients with COVID‐19. Indeed, several studies have proposed and investigated the role of KD in the treatment of SARS‐CoV‐2 infection. For example, Seungjin et al.^5^ revealed that KD restrains aging‐induced exacerbation of coronavirus infection in mice and proposed a harnessing of the ketogenic immunometabolic checkpoint as a potential treatment against coronavirus infection in the aged. Although their results have revealed a positive role for KD in the treatment of SARS‐CoV‐2 infection, the underlying mechanisms remain unclear.

To uncover the underlying mechanisms, Karagiannis et al.^1^ analyzed the metabolic alterations and immune responses in 39 healthy volunteers, 46 patients with moderate COVID‐19, 64 patients with severe COVID‐19, 32 patients with influenza, and 15 patients with bacterial pneumonia. Both patients with moderate and severe COVID‐19 had low serum BHB levels but did not have low glucose or insulin levels, or the total calories supplied, compared with patients with influenza‐associated ARDS. They also observed lower levels of IFN‐related cytokines, including IFN‐γ, IFN‐λ, and IFN‐γ‐induced protein‐10 in patients with COVID‐19 than in patients with influenza. These results indicate impaired infection‐induced ketogenesis and dysregulated T‐cell responses in patients with SARS‐CoV‐2 infection. Although previous studies have demonstrated that KD protects mice from coronavirus infection and that BHB promotes the function of effector and memory CD8^+^ T cells, it is unclear whether ketone bodies have the ability to alter CD4^+^ T‐cell immune responses. To answer this question, the authors performed extracellular flux analysis and single‐cell energetic metabolism by profiling translation inhibition metabolic profiling. The addition of BHB bolstered mitochondrial oxidative phosphorylation (OXPHOS) in human and mouse CD4^+^ T cells, thereby supporting the production of IFN‐γ and tumor necrosis factor alpha. To further investigate the underlying metabolic alterations caused by BHB, the authors used ^13^C‐labeled BHB tracing analysis to investigate the role of BHB in CD4^+^ T‐cell metabolism. ^13^C‐labeled BHB was found not only in tricarboxylic acid cycle intermediates (citrate and malate), but also in bioenergetic amino acids (glutamate and aspartate) and oxidized glutathione, suggesting that BHB acts as an alternative carbon source to fuel OXPHOS and the production of bioenergetic amino acids and glutathione for T cells.

In‐line with recent studies, the authors also found that T cells from patients with severe COVID‐19 displayed metabolic dysregulation and defective effector function, pointing toward a novel mechanism beyond immune overactivation underlying severe COVID‐19. Given the crucial role of BHB in CD4^+^ T‐cell metabolism and function, they hypothesized that BHB supplementation could reprogram CD4^+^ T‐cell metabolism, thereby increasing the antiviral immune response in patients with COVID‐19. This hypothesis was confirmed by the elevated serum BHB levels in mice infected with influenza A virus or SARS‐CoV‐2 and fed a KD (consisting of a 4:1 ratio of fat to proteins and carbohydrates) or supplemented with ketone ester (20 mg/ml) in their drinking water BHB supplementation consistently increased the percentage and total number of functional CD4^+^ T cells in infected mice. Moreover, transcriptional and protein analysis revealed that BHB treatment promoted CD4^+^ T cells to oxidize fatty acids and amino acids, increased IFN‐γ production and viral clearance, and reduced virus‐induced pulmonary fibrosis and damage.

In conclusion, Karagiannis et al.^1^ uncovered a potential link between impaired infection‐induced ketogenesis in COVID‐19 and SARS‐CoV‐2‐induced immune dysregulation, which provides new insights into KD‐based COVID‐19 treatment. In addition, one of the main findings of this study is that BHB can be used as a fuel for CD4/8 T cells to support mitochondrial metabolism during viral infection. However, some important issues remain to be resolved. For example, the underlying reasons why SARS‐CoV‐2‐induced ARDS, but not influenza‐induced ARDS, inhibits infection‐induced ketogenesis remain unknown. In addition to mitochondrial metabolism, whether ketone bodies signaling through specific receptors are an important pathway in T cells or other immune cells remains unknown. Further investigation of these issues may provide a new perspective for understanding the critical role of ketogenesis in immune regulation under physiological or pathological conditions.

## AUTHOR CONTRIBUTIONS

Bin Wang and Lei Zhang contributed equally to this work. Bin Wang and Lei Zhang conceived and drafted the manuscript. Bin Wang drew the figures. Lei Zhang discussed the concepts of the manuscript. Long Zhang and Fangfang Zhou provided valuable discussion and revised the manuscript. All authors have read and approved the article.

## CONFLICT OF INTERESTS

The authors declare no conflict of interest.

## ETHICS STATEMENT

Not applicable.

## Data Availability

Not applicable.
